# Supplementary material for: Pseudomonas and Curtobacterium Strains from Olive Rhizosphere Characterized and Evaluated for Plant Growth Promoting Traits
Source: Plants (Basel). 2022 Aug 29;11(17):2245. doi: 10.3390/plants11172245 (PMC9460707; doi:10.3390/plants11172245)
Supplement: Supplementary file 1 [file plants-11-02245-s001.zip › Figure S1.pdf]

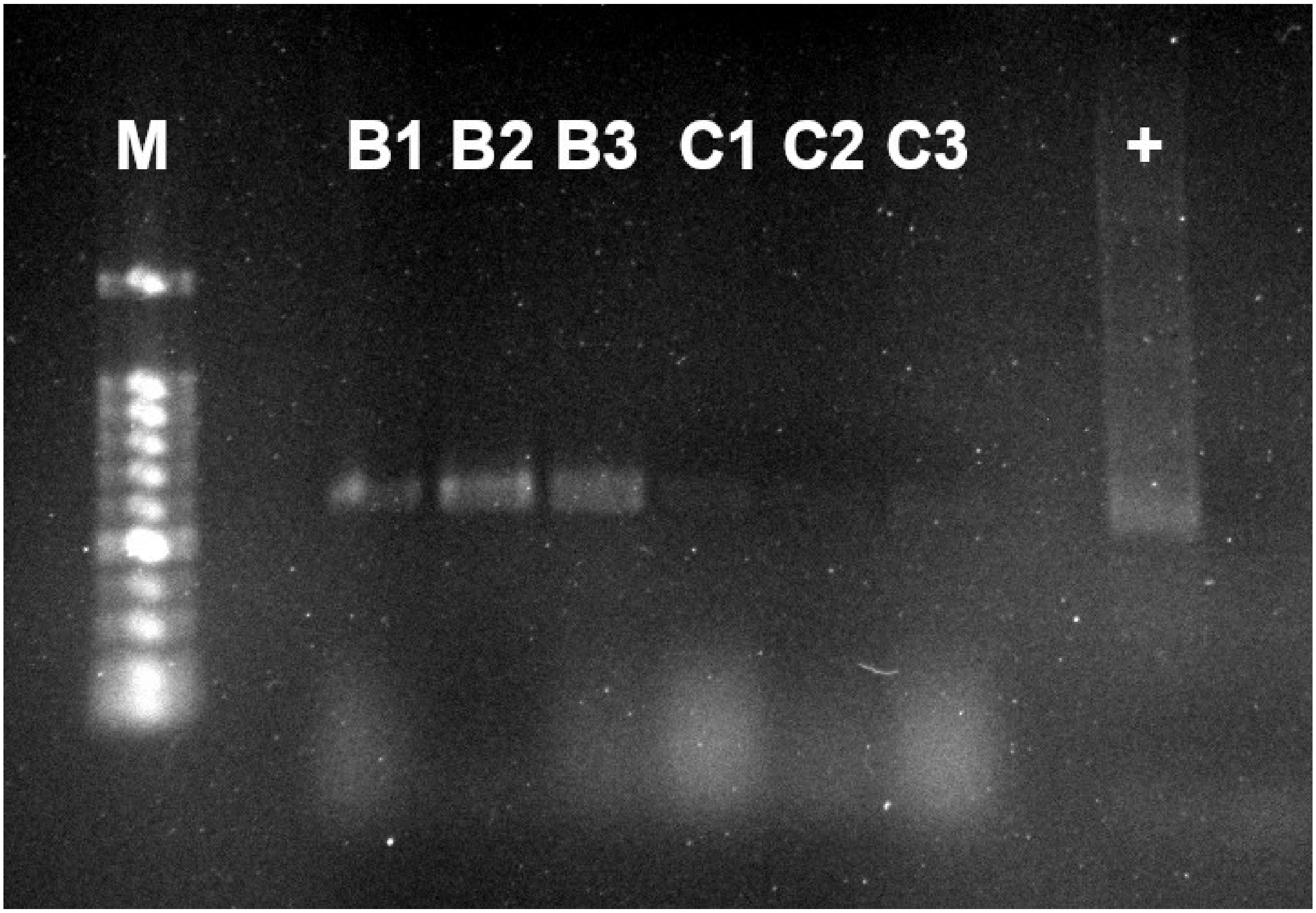

Figure S1. 1% agarose gel showing the PCR products amplified from 16S rRNA gene sequences extracted from randomly chosen samples. B1-3: inoculated samples; C1-3: non inoculated samples; M: 100 bp DNA ladder; +: positive control obtained amplifying DNA extracted from axenic PK18 culture. The faint amplicons from samples C1-3 were sequenced and belong to *Pseudomonas* spp. other than the ones used as inoculants, hence we speculate that they come from to endophyte bacteria.
